# A near-complete genome of the uncultured *Staphylococcus aureus* phage COMBAT-CF_PAR1 isolated from the lungs of an infant with cystic fibrosis

**DOI:** 10.1128/mra.01047-24

**Published:** 2024-10-29

**Authors:** Patricia Agudelo-Romero, Jose A. Caparros-Martin, Abhinav Sharma, Montserrat Saladié, Peter D. Sly, Stephen M. Stick, Fergal O´Gara, COMBAT study group

**Affiliations:** 1 Wal-Yan Respiratory Research Centre, The Kids Research Institute Australia, Perth, Western Australia, Australia; 2 Australian Research Council Centre of Excellence in Plant Energy Biology, School of Molecular Sciences, The University of Western Australia, Perth, Western Australia, Australia; 3 European Virus Bioinformatics Center, Friedrich-Schiller-Universitat Jena, Thuringia, Germany; 4 Curtin Health Innovation Research Institute (CHIRI), Curtin University, Perth, Western Australia, Australia; 5 DSI-NRF Centre of Excellence for Biomedical Tuberculosis Research; SAMRC Centre for Tuberculosis Research; Division of Molecular Biology and Human Genetics, Faculty of Medicine and Health Sciences, Stellenbosch University, Cape Town, South Africa; 6 Children’s Health and Environment Program, Child Health Research Centre, The University of Queensland, Brisbane, Australia; 7 Department of Respiratory and Sleep Medicine, Perth Children’s Hospital, Perth, Western Australia, Australia; 8 Centre for Cell Therapy and Regenerative Medicine, School of Medicine and Pharmacology, The University of Western Australia and Harry Perkins Institute of Medical Research, Perth, Western Australia, Australia; 9 BIOMERIT Research Centre, School of Microbiology, University College Cork, Cork, Ireland; Department of Biology, Queens College, Queens, New York, USA

**Keywords:** *Staphylococcus aureus*, bacteriophage assembly, cystic fibrosis

## Abstract

In cystic fibrosis, bacteria–bacteriophage interaction in the lower airways is poorly understood. We present the near-complete genome of the uncultured Siphovirus-like bacteriophage, *Staphylococcus aureus* phage COMBAT-CF_PAR1, isolated from the lower airways. The genome spans 41,510 bp with 33.45% guanine–cytosine content and contains 65 open reading frames.

## ANNOUNCEMENT

Cystic fibrosis (CF) is a genetic disease characterized by persistent infection and inflammation, leading to irreversible lung damage (1). In CF, a diverse respiratory microbiota progresses to pathogen-dominated communities as individuals age and lung function declines (2, 3). *Staphylococcus aureus* respiratory infections are prevalent in over 50% of children with CF under 2 years old (4). Understanding bacteriophage populations associated with bacterial infections is crucial due to their impact on bacterial dynamics and antibiotic resistance.

We present a near-complete genome of a novel, uncultured endogenous *Staphylococcus aureus* Phage COMBAT-CF_PAR1. This bacteriophage was characterized using shotgun metagenomic data obtained from DNA extracted from bronchoalveolar lavage fluid (BALF) of a CF infant, confirmed positive for *S aureus* through clinical microbiology (5, 6).

This study, aligned with the COMBAT-CF study protocol (Clinicaltrials.gov: NCT01270074), received approval from the site-specific hospital’s Human Research Ethics Committee, with parental/guardian informed consent (6). This ancillary study explored the airway microbiome of the COMBAT-CF BALF samples at 12 months of age collected in 2017 (5, 6). Using a low biomass protocol (7), we extracted microbial DNA from 2 mL of BAL (5). High-quality DNA underwent library preparation using the Nextera XT kit (Illumina, San Diego, CA, USA) and was sequenced on the Illumina NovaSeq 6000 platform by Genewiz (China), using a 150-bp pair-end configuration (137 million reads) (8). The raw FASTQ files were processed through EVEREST-meta v0.1.0 (https://github.com/agudeloromero/EVEREST_meta) (9), using default parameters and database v0.0.3 (10) as we previously described (11).

Following human read removal with minimap2 v2.24 (12), deduplication, and digital normalization steps using BBMAP v38.96 (13), approximately 1.2 million non-human reads were retained for *de novo* assembly with SPAdes v3.13.0 (14). Viral contigs (vContigs) >5,000 bp were retained using VirSorter v2.2.3 (15), followed by a CheckV v 0.9.0 quality genome assessment (16). Additional steps were performed for functional annotation with Pharokka v1.3.0 (17), genome termini using PhageTerm v1.0.11 (18), virulence/resistance gene identification through CARD database (28 July 2019) via ABRICATE v1.0.1 (19, 20), calculation of average nucleotide identity (ANI) by orthoANI v0.5.0 (21), and host prediction using iPhop v1.2.0 (22).

**Fig 1:**
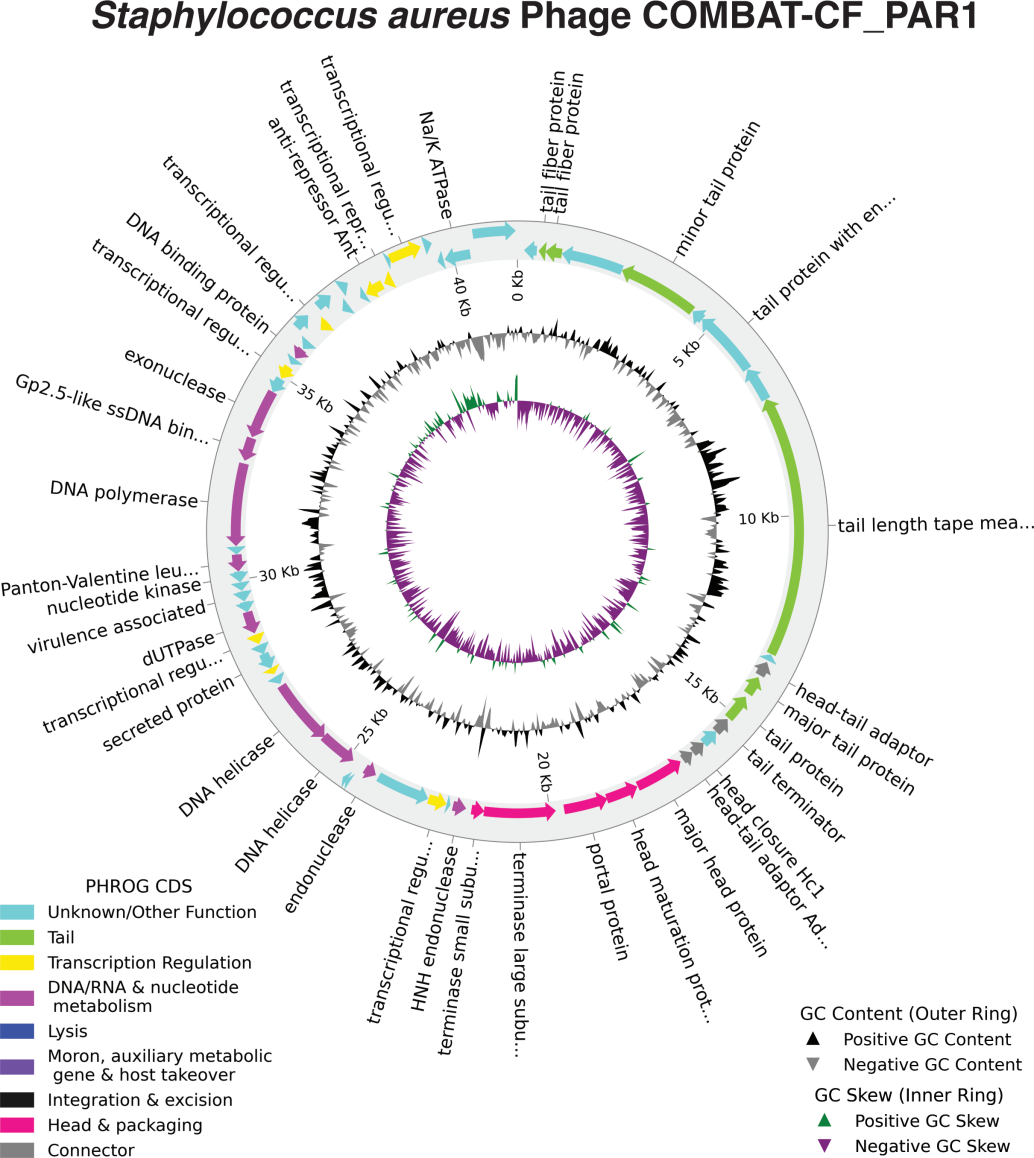
Genome structure of *Staphylococcus aureus* phage COMBAT-CF_PAR. The outer ring depicts the circularized phage genome, with CDS annotated by predicted function. The PHROG CDS within the genome are color coded to summarize different functional categories.

The COMBAT-CF_PAR1 bacteriophage genome spans 41,510 bp (33.45% guanine–cytosine [GC] content), containing 65 predicted open reading frames. We were unable to determine genome termini likely due to the tagmentation step during library preparation (18). No virulence or antimicrobial resistance genes were detected (19, 20). Predicted genes are associated with DNA, RNA, nucleotide metabolism, and transcription regulation (Fig. 1; Table 1).

**TABLE 1:** Genomic features of the draft genome sequence of the uncultured *Staphylococcus aureu*s phage COMBATCF_PAR1, isolated from a pediatric BALF sample in a CF patient

Features	*Staphylococcus aureus* phage COMBAT-CF_PAR1
Genome size (bp)	41,510
Genome coverage (RPKM)	11,420.5921
No. of reads	15,247
Coverage (X)	55.94
Breadth of coverage %	100
GC content (%)	33.45
CheckV quality (%)	High quality
CheckV completeness (%)	91.23
CDS	65
Connector	3
DNA, RNA, and nucleotide metabolism	10
Head and packaging	5
Integration and excision	0
Lysis	0
Moron, auxiliary metabolic gene, and host takeover	0
Others	5
Tail	6
Transcription regulation	8
Unknown function	28
tRNAs, CRISPRs, tmRNAs	0
Virulence factors (VFDB)	0
AMR genes (CARD database; 28 July 2019)	0
Lowest common ancestor, from order to genus (NCBI)	Siphovirus-like
Closest related phage NBCI nt (GenBank accession no.)	*Staphylococcus* phage phiSauS-IPLA35 (NC_011612.1; 45,344-bp long)
ANI similarity (%)	97.92
Lowest common ancestor, from order to genus (UNIPROT)	Siphovirus-like
Closest related phage UNIPROT aa (UniProtKB accession No.)	*Staphylococcus* phage Sa2wa_st8 (A0A514U6D1)
Baltimore	Group I (dsDNA)
Host prediction, genus level	*Staphylococcus*
Confidence score of host prediction	100

For taxonomic classification, EVEREST employs the MMseqs2 taxonomy tool (MMSeqs2 v13.45111) (23, 24), leveraging NCBI (nucleotide) and UNIPROT (amino acid) viral databases (2023). Both databases classified COMBAT-CF_PAR1 as Siphovirus-like. *Staphylococcus phage* (NC_011612.1) emerged as the closest related bacteriophage, with a genome that is 3,834 bp longer (Table 1). This result was validated by calculating the average nucleotide identity (ANI) (21) with *Staphylococcus phage* (NC_011612.1), indicating a 97.92% similarity, and host prediction supported this conclusion (22).

## Data Availability

This project has been deposited in the Sequence Read Archive SRR29469209, BioProject PRJNA1126024, BioSample SAMN40747810, GeneBank accession PP961382.

## AFTER EPUB

*[This article was published on 29 October 2024 with errors in an affiliation. The fifth affiliation was corrected in the current version, posted on 15 November 2024.]*

## References

[1] Elborn JS. 2016. Cystic fibrosis. The Lancet 388:2519–2531. doi:10.1016/S0140-6736(16)00576-6 27140670

[2] Cuthbertson L, Walker AW, Oliver AE, Rogers GB, Rivett DW, Hampton TH, Ashare A, Elborn JS, De Soyza A, Carroll MP, Hoffman LR, Lanyon C, Moskowitz SM, O’Toole GA, Parkhill J, Planet PJ, Teneback CC, Tunney MM, Zuckerman JB, Bruce KD, van der Gast CJ. 2020. Lung function and microbiota diversity in cystic fibrosis. Microbiome 8:45. doi:10.1186/s40168-020-00810-3 32238195 PMC7114784

[3] Frayman KB, Armstrong DS, Carzino R, Ferkol TW, Grimwood K, Storch GA, Teo SM, Wylie KM, Ranganathan SC. 2017. The lower airway microbiota in early cystic fibrosis lung disease: a longitudinal analysis. Thorax 72:1104–1112. doi:10.1136/thoraxjnl-2016-209279 28280235

[4] Rumpf C, Lange J, Schwartbeck B, Kahl BC. 2021. Staphylococcus aureus and cystic fibrosis-a close relationship. What can we learn from sequencing studies? Pathogens 10:1177. doi:10.3390/pathogens10091177 34578208 PMC8466686

[5] Caparrós-Martín JA, Saladie M, Agudelo-Romero SP, Reen FJ, Ware RS, Sly PD, Stick SM, O’Gara F, COMBAT study group. 2023. Detection of bile acids in bronchoalveolar lavage fluid defines the inflammatory and microbial landscape of the lower airways in infants with cystic fibrosis. Microbiome 11:132. doi:10.1186/s40168-023-01543-9 37312128 PMC10262387

[6] Stick SM, Foti A, Ware RS, Tiddens H, Clements BS, Armstrong DS, Selvadurai H, Tai A, Cooper PJ, Byrnes CA, Belessis Y, Wainwright C, Jaffe A, Robinson P, Saiman L, Sly PD. 2022. The effect of azithromycin on structural lung disease in infants with cystic fibrosis (COMBAT CF): a phase 3, randomised, double-blind, placebo-controlled clinical trial. Lancet Respir Med 10:776–784. doi:10.1016/S2213-2600(22)00165-5 35662406

[7] Saladié M, Caparrós-Martín JA, Agudelo-Romero P, Wark PAB, Stick SM, O’Gara F. 2020. Microbiomic analysis on low abundant respiratory biomass samples; improved recovery of microbial DNA From bronchoalveolar lavage fluid. Front Microbiol 11. doi:10.3389/fmicb.2020.572504 PMC7573210 33123104

[8] Andrews S, Krueger F, Segonds-Pichon A, Biggins L, Krueger C, Wingett S. 2012. FastQC Babraham Institute. Babraham, UK

[9] Agudelo-Romero P, Conradie T, Kicic A, Caparros-Martin J, Stick S. 2024. EVEREST-Meta: a pipEline for viral assEmbly and chaRactEriSaTion for METAgenomics

[10] Agudelo-Romero P, Sharma A, Conradie T, Kicic A, Caparros-Martin JA, Stick SM. 2023. Database for EVEREST (pipEline for Viral assEmbly and chaRactEriSaTion)

[11] Conradie T, Caparros-Martin JA, Egan S, Kicic A, Koks S, Stick SM, Agudelo-Romero P. 2024. Exploring the complexity of the human respiratory virome through an in silico analysis of shotgun metagenomic data retrieved from public repositories. Viruses 16:953. doi:10.3390/v16060953 38932245 PMC11209621

[12] Li H. 2018. Minimap2: pairwise alignment for nucleotide sequences. Bioinformatics 34:3094–3100. doi:10.1093/bioinformatics/bty191 29750242 PMC6137996

[13] Bushnel l B. 2016. BBMAP: short-read aligner, and other bioinformatics tools

[14] Prjibelski A, Antipov D, Meleshko D, Lapidus A, Korobeynikov A. 2020. Using SPAdes De Novo assembler. Curr Protoc Bioinforma 70:e102. doi:10.1002/cpbi.102 32559359

[15] Guo J, Bolduc B, Zayed AA, Varsani A, Dominguez-Huerta G, Delmont TO, Pratama AA, Gazitúa MC, Vik D, Sullivan MB, Roux S. 2021. VirSorter2: a multi-classifier, expert-guided approach to detect diverse DNA and RNA viruses. Microbiome 9:37. doi:10.1186/s40168-020-00990-y 33522966 PMC7852108

[16] Nayfach S, Camargo AP, Schulz F, Eloe-Fadrosh E, Roux S, Kyrpides NC. 2021. CheckV assesses the quality and completeness of metagenome-assembled viral genomes. Nat Biotechnol 39:578–585. doi:10.1038/s41587-020-00774-7 33349699 PMC8116208

[17] Bouras G, Nepal R, Houtak G, Psaltis AJ, Wormald PJ, Vreugde S. 2023. Pharokka: a fast scalable bacteriophage annotation tool. Bioinformatics 39:btac776. doi:10.1093/bioinformatics/btac776 36453861 PMC9805569

[18] Garneau JR, Depardieu F, Fortier L-C, Bikard D, Monot M. 2017. PhageTerm: a tool for fast and accurate determination of phage termini and packaging mechanism using next-generation sequencing data. Sci Rep 7:8292. doi:10.1038/s41598-017-07910-5 28811656 PMC5557969

[19] Jia B, Raphenya AR, Alcock B, Waglechner N, Guo P, Tsang KK, Lago BA, Dave BM, Pereira S, Sharma AN, Doshi S, Courtot M, Lo R, Williams LE, Frye JG, Elsayegh T, Sardar D, Westman EL, Pawlowski AC, Johnson TA, Brinkman FSL, Wright GD, McArthur AG. 2017. CARD 2017: expansion and model-centric curation of the comprehensive antibiotic resistance database. Nucleic Acids Res 45:D566–D573. doi:10.1093/nar/gkw1004 27789705 PMC5210516

[20] Seemann T. 2020. Mass screening of contigs for antimicrobial resistance or virulence genes. https://github.com/tseemann/abricate.

[21] Yoon S-H, Ha S-M, Lim J, Kwon S, Chun J. 2017. A large-scale evaluation of algorithms to calculate average nucleotide identity. Antonie Van Leeuwenhoek 110:1281–1286. doi:10.1007/s10482-017-0844-4 28204908

[22] Roux S, Camargo AP, Coutinho FH, Dabdoub SM, Dutilh BE, Nayfach S, Tritt A. 2023. iPHoP: an integrated machine learning framework to maximize host prediction for metagenome-derived viruses of archaea and bacteria. PLoS Biol 21:e3002083. doi:10.1371/journal.pbio.3002083 37083735 PMC10155999

[23] Mirdita M, Steinegger M, Breitwieser F, Söding J, Levy Karin E. 2021. Fast and sensitive taxonomic assignment to metagenomic contigs. Bioinformatics 37:3029–3031. doi:10.1093/bioinformatics/btab184 33734313 PMC8479651

[24] Steinegger M, Söding J. 2017. MMseqs2 enables sensitive protein sequence searching for the analysis of massive data sets. Nat Biotechnol 35:1026–1028. doi:10.1038/nbt.3988 29035372

